# The Interplay between Drug and Sorbitol Contents Determines the Mechanical and Swelling Properties of Potential Rice Starch Films for Buccal Drug Delivery

**DOI:** 10.3390/polym13040578

**Published:** 2021-02-15

**Authors:** Bilal Harieth Alrimawi, May Yee Chan, Xin Yue Ooi, Siok-Yee Chan, Choon Fu Goh

**Affiliations:** Discipline of Pharmaceutical Technology, School of Pharmaceutical Sciences, Universiti Sains Malaysia, Minden 11800, Penang, Malaysia; bilalrimawe@student.usm.my (B.H.A.); peace_mayyee12@hotmail.com (M.Y.C.); yuesone@gmail.com (X.Y.O.); sychan@usm.my (S.-Y.C.)

**Keywords:** rice starch, paracetamol, plasticiser, antiplasticisation, mechanical, water absorption capacity, elongation to break

## Abstract

Rice starch is a promising biomaterial for thin film development in buccal drug delivery, but the plasticisation and antiplasticisation phenomena from both plasticisers and drugs on the performance of rice starch films are not well understood. This study aims to elucidate the competing effects of sorbitol (plasticiser) and drug (antiplasticiser) on the physicochemical characteristics of rice starch films containing low paracetamol content. Rice starch films were prepared with different sorbitol (10, 20 and 30% *w*/*w*) and paracetamol contents (0, 1 and 2% *w*/*w*) using the film casting method and were characterised especially for drug release, swelling and mechanical properties. Sorbitol showed a typical plasticising effect on the control rice starch films by increasing film flexibility and by reducing swelling behaviour. The presence of drugs, however, modified both the mechanical and swelling properties by exerting an antiplasticisation effect. This antiplasticisation action was found to be significant at a low sorbitol level or a high drug content. FTIR investigations supported the antiplasticisation action of paracetamol through the disturbance of sorbitol–starch interactions. Despite this difference, an immediate drug release was generally obtained. This study highlights the interplay between plasticiser and drug in influencing the mechanical and swelling characteristics of rice starch films at varying concentrations.

## 1. Introduction

In recent years, advances in thin film technology have broadened the repertoire for drug delivery, especially through the buccal route [1,2,3,4]. An ideal film for buccal drug delivery should facilitate the drug release and absorption [5]. In addition, the films should possess adequate flexibility and strength in order to tolerate stress upon application into the buccal cavity and to achieve a desired drug release [6,7,8]. The flexibility and strength of buccal films are immensely affected by the type of film-forming agents used, which are usually polymers [9]. The key characteristics of buccal films including drug release profiles, mucoadhesive properties and mechanical strength can be governed by adjusting the composition of polymers [10]. 

Rice starch is a biopolymer widely used to produce biodegradable films as a replacement for plastic packaging [11,12,13]. The formation of films from biomaterials such as starch and proteins as a film-forming agent usually requires a plasticiser [14]. Plasticisers are necessary to overcome film brittleness owing to the high polymer intermolecular forces [15]. The incorporation of plasticisers also enhances the mechanical properties and flexibility of the film [16,17,18], the water vapour permeability [19] and the film swelling [20,21,22]. The attractive characteristics of plasticised rice starch films are imperative and essential for development as a functional biopolymer film for oromucosal preparations such as buccal films [7].

The plasticisation effect on different properties of starch-based films is usually concentration-dependent [23,24,25,26,27]. The typical functions of a plasticiser are well reflected in its ability to improve the film mechanical and physical properties such as swelling behaviour. However, it is interesting to note that plasticisers may exert an antiplasticising effect, usually at a concentration exceeding the maximal limit of plasticising action [28]. An increased concentration of glycerol and sorbitol exceeding 60% *w*/*w* causes a drop in the swelling behaviour of sorghum starch films [21] and sugar palm starch films [29]. Muscat et al. [30] reported that the incorporation of glycerol or xylitol at a concentration of 15% *w*/*w* resulted in a brittle film despite having a high tensile strength. Moreover, Sanyang, Sapuan, Jawaid, Ishak and Sahari [25] observed a considerable reduction in flexibility of sugar palm films when glycerol content was added beyond 45% *w*/*w*. 

The unconventional antiplasticisation effect is mostly reported with the use of low plasticiser concentrations, and this phenomenon can be engineered to modulate the mechanical and physical properties of polymeric systems, usually in food science. In particular, the antiplasticisation phenomenon is given minimal attention and is not well recognised in pharmaceutical formulation to fully comprehend the basics of plasticiser–polymer interactions. The earlier formulation work by Lachman and Drubulis [31], in 1964, and by Guo [32], in 1993, are among the pioneer studies documenting the antiplasticisation of various plasticisers on polymeric films. Many years later, concern about the antiplasticisation effect has resurfaced and was highlighted in a report by Chamarthy and Pinal [33] to emphasise the antiplasticising effect of sorbitol in governing the release of theophylline from starch-based hot melt extrudates. The phenomenon was later found in several reports, and it is interesting to note that active pharmaceutical ingredients can exert the same antiplasticisation action in polymeric systems, especially at low concentrations [34,35]. 

In our previous study, plasticiser-influenced swelling behaviour and drug crystallinity within the polymeric matrices were identified as the major contributing factors in governing the drug release performance from the rice starch films [36]. Even though two different plasticisers—glycerol and sorbitol—were used, a complete understanding of their plasticisation effects on the performance of the rice starch films was limited due to the use of a single plasticiser concentration and the influence of drug crystallinity at high drug concentration. In addition, this previous work did not report mechanical data.

Following this, the current study magnifies the role of the plasticiser by applying a selected plasticiser at varying concentrations to gain further knowledge related to the plasticisation action on rice starch films. At the same time, a low drug content is incorporated to examine the antiplasticisation action, which was not widely explored apart from minimising the influence of drug crystallinity on the observations. Therefore, we aim to investigate the competing effects of plasticisers and drugs (antiplasticisers) on the mechanical properties, swelling behaviour and drug release profiles of rice starch films containing low paracetamol (PCM) content. Sorbitol was selected for the current work due to its stronger interaction with rice starch and influence on the drug dissolution studies compared with glycerol reported in the previous study [36]. Sorbitol was also found to possess a superior effect over glycerol on the mechanical properties of films prepared from corn starch [37] and potato starch [38].

## 2. Materials and Methods

### 2.1. Materials

The rice grains were obtained from Sekinchan, Sabak Bernam, Selangor, Malaysia, and the rice starch was extracted as documented in our previous work [36]. The total starch content determined using the total starch assay kit (Megazyme International, Bray, Ireland) is 84.3 g/100 g rice starch. The amylose content of the rice starch estimated using an iodometric method [39] is 20.8 g/100 g rice starch, while the amylopectin content (difference between total starch and amylose content) is 63.5 g/100 g rice starch. PCM was obtained from Euro Chemo-Pharma, Perai, Penang, Malaysia. Sorbitol was obtained from R&M Chemicals, London, UK. Polyvinyl alcohol (PVA) (molecular weight = 115,000) was obtained from VWR^®^ International, Radnor, PA, USA. To prepare simulated saliva fluid, 2.38 g of disodium hydrogen phosphate, 0.19 g monopotassium phosphate and 8 g of sodium chloride were dissolved in 1 L of distilled water, and the solution pH was adjusted to 6.80 by 1 M hydrochloric acid. All other reagents were of analytical grade.

### 2.2. Preparation of Rice Starch Films

The film casting method was modified from our previous work to prepare rice starch films with different compositions of sorbitol and PCM [36]. A mixture containing 1.8 g of starch and 0.2 g of PVA were prepared in 50 mL distilled water with continuous stirring for 10 min at room temperature until a homogeneous dispersion was formed. PVA was added to improve the peeling of rice starch films. Sorbitol was added at 0.2, 0.4 and 0.6 g in addition to the mixture, which corresponded to 10, 20 and 30% *w*/*w* of sorbitol based on the weight of the starch–PVA mixture. The dispersion was stirred at 90 °C for 2 h before pouring into a polypropylene container (24 × 7 cm^2^). The film was dried at 50 °C overnight (18–20 h) and kept in a desiccator. Drug-loaded films were prepared by adding PCM in a similar manner to that of sorbitol to the starch mixture that corresponded to 1 and 2% *w*/*w* of PCM on the weight basis of the starch–PVA mixture prior to heating. Drug-free (control) films were coded as CS with a number representing the percentage of sorbitol. While drug-loaded films were coded as S for sorbitol and P for PCM with a number following to represent their concentration added. For instance, S10P1 refers to rice starch films containing 10% *w*/*w* of sorbitol and 1% *w*/*w* of PCM.

### 2.3. Characterisation of Rice Starch Films

#### 2.3.1. Film Thickness

Films thickness was measured using a Mitutoyo micrometer M820-25 (measuring range: 0–25 mm; scale interval: 0.001 mm). The average thickness was determined from the measurement of 10 random spots on the film.

#### 2.3.2. Water Content

Water content was determined in triplicates from the differential weight before and after drying a film with a size of ≈1 cm^2^ (weight: 0.02–0.03 g) on a hot plate at 120 °C for 2 h. 

#### 2.3.3. Drug Loading Efficiency 

Drug distribution uniformity was tested by the analysing drug content at various locations on the film. Film samples (≈4 cm^2^) were immersed into 15 mL simulated saliva fluid at 37.0 ± 0.5 °C with constant stirring at 500 rpm for 24 h. A total of 10 mL of sample was withdrawn after 24 h and analysed in triplicate at 243 nm using PerkinElmer Lambda XLS UV/VIS spectrophotometer. Drug loading efficiency was calculated using Equation (1):

(1) $$\mathrm{Drug}\;\mathrm{loading}\;\mathrm{efficiency}\;\left(\%\right)=\frac{\mathrm{Amount}\;\mathrm{of}\;\mathrm{drug}\;\mathrm{detected}}{\mathrm{Amount}\;\mathrm{of}\;\mathrm{drug}\;\mathrm{loaded}}\times 100\%$$

#### 2.3.4. Water Absorption Capacity and Kinetics

Swelling behaviour was determined by evaluating water absorption capacity (WAC). This was performed by immersing a mesh basket containing a film (≈4 cm^2^) in simulated saliva fluid at 37.0 ± 0.5 °C with constant stirring at 100 rpm over 3 h. The basket containing films was removed at predetermined intervals (5, 10, 15, 30, 45, 60, 120 and 180 min), and excess fluid on the surface of the film was wiped off gently using tissue paper before weighing. The WAC was determined as g water/g sample in triplicates using Equation (2) [36]:

(2) $$Water\;absorption\;capacity\;\left(WAC\right)=\frac{\left(W_{t}-W_{0}\right)}{W_{0}}$$

where *W_t_* is the film weight at time *t* and *W_t_* refers to the initial film weight.

The water absorption (swelling) kinetic of rice starch films was determined based on the Peleg model using Equation (3) [40,41]:

(3) $$M_{t}=M_{0}+\frac{t}{k_{1}+k_{2}t}$$

where *M_t_* is the film weight at time *t*, *M*_0_ is the initial film weight, *k*_1_ is Peleg rate constant and *k*_2_ is Peleg capacity constant.

#### 2.3.5. Mechanical Properties 

**Figure 1:**
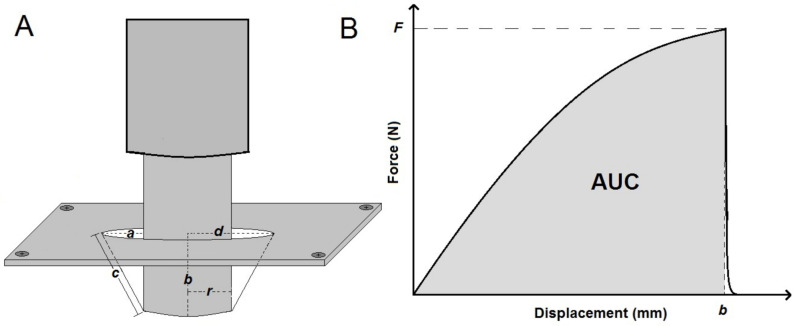
(**A**) Determination of elongation to break: sample deformation before break (*d* = radius of the rice starch film in the sample holder opening (initial length); *a* = initial length − radius of probe; *b* = displacement of the probe; *c* + *r* = length after strain; *c* = length of a after strain; and *r* = radius of probe). (**B**) Typical plot of force versus displacement for films subjected to puncture. *F* is the force required to puncture the film, and *b* is the distance at break.

The puncture strength (PS), elongation to break (EB) and energy to puncture (EP) were determined using texture analyser TA-XT plus following the previous method described by Preis et al. [37]. A film (2 × 2 cm^2^) was fixed by four screws between two plates with a hole of 10 mm diameter and an area of 78.45 mm^2^. This hole was centred directly under a metal cylindrical probe with a flat-faced surface (diameter: 5 mm). The probe was adjusted to move towards the hole with a velocity of 1 mm/s. The 5 kg load cell system had a trigger sensitivity of 0.001 N. The system started reading the displacement and force once the probe contacted the sample surface and continued reading until the film broke apart. The puncture properties were then calculated; Figure 1A illustrates the film elongation by the probe, which was calculated by applying Equation (4):

(4) $$Elongation\;to\;break\;\left(EB\right)=\left(\frac{\sqrt{a^{2}+b^{2}}+r}{d}-1\right)\times \;100$$

where *d* is the radius of the film sample before the test, *b* is the penetration depth or vertical displacement by the probe, *r* is the radius of the probe and *a* refers to the difference between the film and the probe radiuses (*a* = *d* − *r*). 

A force–displacement plot (Figure 1B) was used to calculate PS and EP of each rice starch film according to Equations (5) and (6), respectively.

(5) $$Puncture\;strength\;\left(PS\right)=\frac{F}{area}$$

where *F* is the maximum applied force recorded during strain and the area is the probe contact area, which is 19.63 mm^2^.

(6) $$Energy\;to\;puncture\;\left(EP\right)=\;\frac{AUC}{volume}$$

where *AUC* (area under the curve) is divided by the total volume of the sample [42].

#### 2.3.6. Attenuated Total Reflectance—Fourier Transform Infrared (ATR–FTIR) Spectroscopy

ATR–FTIR spectroscopic spectra of rice starch films were recorded using Nicolet Fourier Transform FTIR-Is 10 model with an ATR diamond crystal over a wavenumber range of 4000–500 cm^−1^. A total of 32 scans were taken for each spectrum, with a resolution of 4 cm^−1^. The spectra obtained were analysed and presented using Essential FTIR V3.50.

#### 2.3.7. In Vitro Drug Dissolution Study

In vitro drug dissolution of rice starch films containing PCM was studied using a calibrated Varian VK7000 Dissolution Apparatus [36]. The vessel was filled with 500 mL of simulated saliva fluid at 37 ± 0.5 °C, with the paddle stirring at 50 rpm. Films (≈1 cm^2^) were placed into a mesh basket before immersing into the dissolution medium. A volume of 10 mL of sample was withdrawn from the dissolution medium and replaced with 10 mL of fresh medium at predetermined periods (2, 5, 7, 10, 15 and 30 min). Each sample was filtered through a cellulose acetate membrane (0.45 μm). The samples were diluted and analysed using a UV spectrophotometer at 243 nm in triplicates. The drug dissolution profiles were corrected based on the drug loading efficiency study.

## 3. Results 

### 3.1. Film Thickness, Water Content and Drug Loading Efficiency 

Table 1 shows the film thickness, water content and drug loading efficiency of rice starch films. Overall, the rice starch films have a thickness of approximately 0.1 mm. The results show increased film thickness with an increase in the sorbitol content in the rice starch films. This result is consistent with the report by Laohakunjit and Noomhorm [43], who reported that rice starch film thickness was increased by the addition of sorbitol. The increased thickness of plasticised films may be explained by the role of plasticisers that increase the free volume in the polymeric matrix, which alters the intermolecular bonds between polymer chains. This leads to an expanded structure and thicker polymer film [41,44]. This observation is similar to a few studies highlighting the role of sorbitol in plasticising starch films [44,45,46]. The incorporation of PCM into the rice starch films causes a reduction in film thickness compared with the corresponding control films. 

Most of the rice starch films contain 0.4–1% of water, with CS30 film having the highest water content among all rice starch films. The effect of sorbitol on the water content was also reported as not significant by several studies [45,46,47]. Generally, the results showed a decreased water content in the drug-loaded films compared to the corresponding control films. However, it is difficult to identify a particular trend for the impact of the concentration of sorbitol on the water content of the films. 

A total of ≈67–93% of drug content was recovered from the drug-loaded rice starch films. Apart from the possibility of uneven drug distribution in the rice starch films, sorbitol released may reduce the solubility of PCM significantly [48]. Thus, this may contribute to underestimation of the drug recovery percentage. 

### 3.2. Water Absorption Capacity and Kinetics

**Figure 2:**
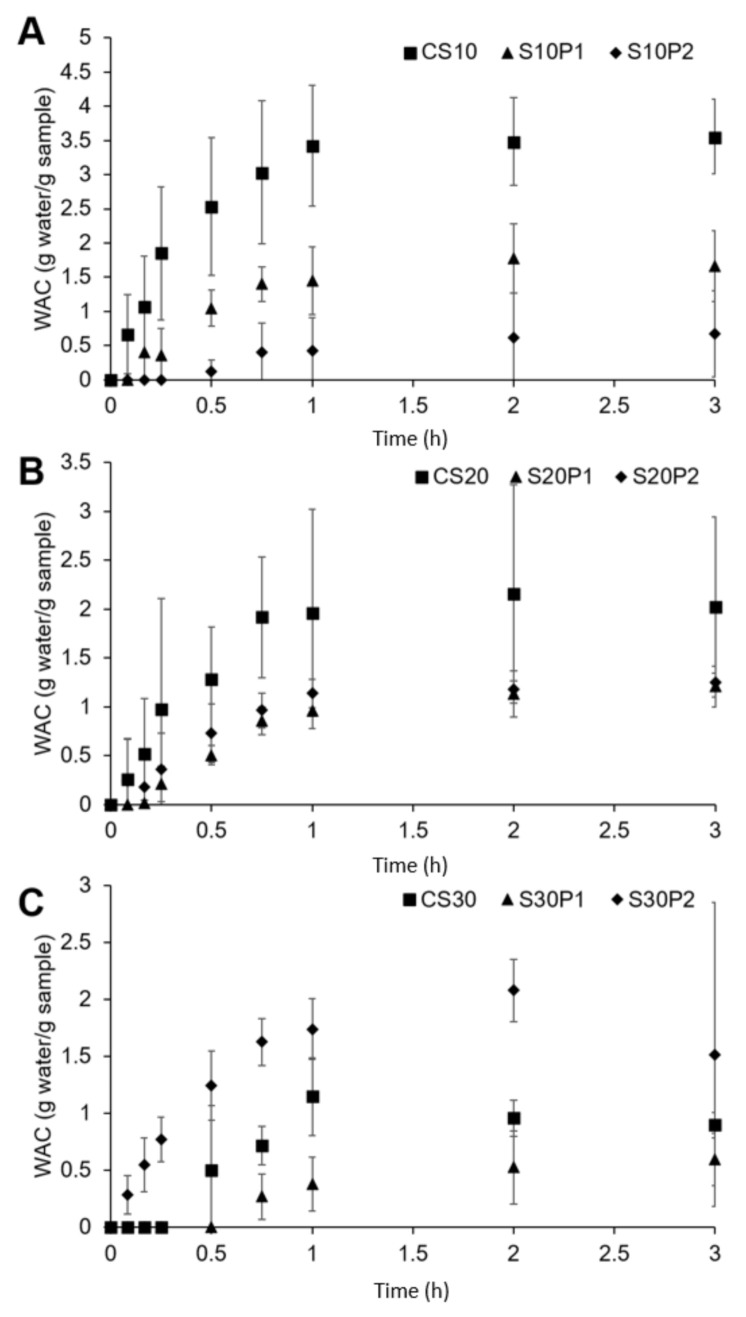
Comparisons of water absorption capacity (WAC) profiles of rice starch films at (**A**) 10, (**B**) 20 and (**C**) 30% *w*/*w* of sorbitol with different drug loadings (n = 3, mean ± SD).

Figure 2 demonstrates the WAC of rice starch films over 3 h. The WAC of control rice starch films reduces with sorbitol content, indicating a reduced swelling ability by sorbitol. Owing to a similar molecular structure between sorbitol and glucose units of the starch polymer, the strong interaction created restricts water from entering the films and results in lower swelling in these films. This is in agreement with past studies on sorbitol-plasticised starch films prepared from sugar palm [45] and sorghum [21]. 

When comparing to the control rice starch films, the rice starch films with 1% *w*/*w* of PCM showed a lower WAC profile. This indicates that PCM reduces the film swelling. When the drug loading increases to 2% *w*/*w*, the WAC increases with sorbitol content. The S20P2 film achieved a similar WAC profile to that of the S20P1 film (Figure 2B), while the S30P2 film surpassed the WAC of both the CS30 and S30P1 films (Figure 2C). This unusual trend suggests that PCM at a higher concentration may assist water absorption and thus exerts a positive effect on film swelling. Nevertheless, a drop in WAC was noticed in most films at 2 h onwards, indicating film erosion. A huge variability in WAC was also found, especially after 2 h due to the fragility of swollen films.

To follow the swelling kinetics, the WAC (before film erosion) was fitted using the Peleg model, as shown in Table 2. The Peleg rate constant (*k*_1_) represents the mass transport rate; the higher *k*_1_, the lower the initial water absorption rate [49]. In contrast, the Peleg capacity constant (*k*_2_) shows the maximum water absorption capacity; the higher the *k*_2_, the lower the absorption capacity.

Control rice starch films plasticised with the highest sorbitol content have higher *k*_1_ and *k*_2_ values, indicating that these films swelled slower and absorbed less water. The addition of PCM at 1% *w*/*w* increased both the *k*_1_ and *k*_2_ values and suggested a reduction in the swelling rate and capacity. However, at 2% *w*/*w* of drug loading, both *k*_1_ and *k*_2_ values reduced with an increased sorbitol content. These results demonstrated that the surplus amount of PCM improved the water absorption rate and capacity.

### 3.3. Mechanical Properties

Table 3 shows the mechanical assessment of all rice starch films. Buccal films were previously suggested by Preis et al. [50] to achieve a PS of at least 0.06 N/mm^2^ using the current test setup. Despite no specific range of film flexibility recommended, EB at 1–33% has been reported by Preis, Knop and Breitkreutz [50]. In our work, both PS (0.2–1.6 N/mm^2^) and flexibility (1–19%) for all drug-loaded rice starch films fall within the ranges reported. Hence, this study demonstrated the crucial role of sorbitol as a functioning material in improving the PS and flexibility of rice starch films.

For control rice starch films, all three mechanical parameters increased with sorbitol concentration. The results indicated that sorbitol content is important in increasing both mechanical characteristics and flexibility of the films. However, this result is different from previous studies that showed a reduced tensile strength and an increased EB of various starch films with sorbitol content [26,38,43,51,52]. These reports collectively suggested that the interference of starch polymer chains and hydrogen bonding due to sorbitol is the reason for a poor tensile strength. The difference in the observations is due to the measurement mode and instrumental setup, as discussed by Radebaugh, Murtha, Julian and Bondi [42]. Unlike the tensile test, the puncture action relies on the contribution of the elongation to break the film until it fails. Therefore, the puncture test can provide a better differentiation of the elongation property than the tensile test. It is not surprising to observe a high PS and EP in this case simply due to the high EB involved. The improved film flexibility is a result of the disturbance of the intermolecular bonds between amylose and amylopectin of the starch polymer matrix by sorbitol that allows for more polymer chain mobility [26,38]. 

With the addition of PCM, similar observations were reported, but a higher EB and EP was recorded for the rice starch films with sorbitol content of 20% *w*/*w* and above compared with the control films. When comparing the mechanical parameters (namely, EB and EP) at a specific sorbitol content, a complex phenomenon was observed. At 10% *w*/*w* of sorbitol, the addition of PCM reduced the values of these mechanical parameters compared with the control films. A similar extent of reduction in these mechanical parameters was observed regardless of the drug loadings. This suggests that the presence of drug molecules in the polymeric matrix may weaken the intermolecular bonds of the starch polymer chains. However, when the sorbitol content doubled, both EB and EP increased with the addition of 1% *w*/*w* of PCM. The plasticising action from the extra sorbitol content may surpass the mechanical weakening by PCM. At 2% *w*/*w* of PCM, EP reduced while EB remained unchanged. This observation strongly suggested that additional PCM can overcome the plasticising action of sorbitol. A similar observation was observed at the highest sorbitol concentration but a decrease in both parameters was noticed at 2% *w*/*w* of PCM. 

### 3.4. ATR-FTIR Spectroscopy

**Figure 3:**
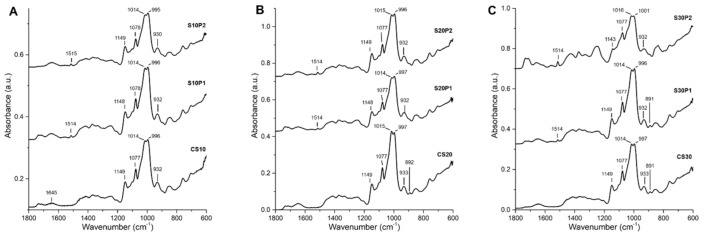
FTIR spectra of rice starch films at (**A**) 10, (**B**) 20 and (**C**) 30% *w*/*w* of sorbitol with different drug loadings.

Figure 3 shows the FTIR spectra of control and drug-loaded plasticised rice starch films at different sorbitol levels. The spectra of control films shared similar peaks including O–H bending vibrations of tightly bound water molecules at 1645 cm^−1^ and several bands mainly related to the C–O stretching vibrations of glucose ring of rice starch in the region of 1240–1077 cm^−1^. These vibrations include CH_2_OH-related modes, C–O–H deformation, C–O–C antisymmetric bridge stretching, C–O–C vibrations, C–O–H stretching and antisymmetric in-plane ring stretching [53]. The peak at 1014 cm^−1^ is attributed to C–O–H solvated. The shifting of C–O stretching vibration to a lower wavenumber at 1014 cm^−1^ was previously reported due to the interaction between the glucose ring of rice starch and sorbitol [36]. The band at 996 cm^−1^ refers to the hydroxyl group on carbon 6 of the glucose ring that can establish intramolecular hydrogen bonding [53]. A band with maxima at 892 cm^−1^ detected in the spectra of CS20 and CS30 films is due to the in-plane bending vibrations of O–H bonds of sorbitol [54]. However, this peak was absent in the spectrum of CS10 film probably due to a low sorbitol concentration.

When PCM was incorporated into the rice starch films, the FTIR spectra were similar to the spectra of the respective control films except with an additional peak at 1514 cm^−1^ referring to the C–N stretching vibrations of PCM (Figure 3B,C). The distinctive features of crystalline PCM were absent in all drug-loaded films, including multiple peaks at 1258, 1242 and 1225 cm^−1^ as well as double peaks at 837 and 796 cm^−1^ due to the C–O and C–N stretching vibrations, respectively (data not shown). This is not surprised as the peaks corresponding to the crystalline PCM were also absent in the sorbitol-plasticised rice starch film with 10% *w*/*w* of PCM, which was confirmed with X-ray diffraction analysis [36]. Specifically, for S30P2 film, several peak shifts at 1143, 1016 and 1001 cm^−1^ related to the C–O stretching vibrations were noticed. This suggests possible interactions of PCM with glucose ring (C–O–H and C–O–C) of rice starch. The appearance of a band at 837 cm^−1^ indicates the presence of PCM given a higher drug loading. 

### 3.5. In Vitro Drug Dissolution Study

**Figure 4:**
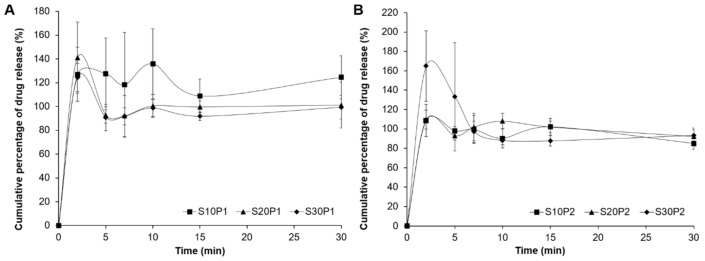
Drug dissolution profiles of rice starch films containing (**A**) 1 and (**B**) 2% *w*/*w* of paracetamol (PCM) with different concentrations of sorbitol (n = 3, mean ± SD).

Figure 4 illustrates the in vitro drug dissolution profile of all rice starch films in 30 mins (corrected based on the drug loading efficiency study). In this study, most rice starch films showed a burst drug release in the first 5–10 min. The huge cumulative percentage of drug release in the beginning can be due to inappropriate homogenisation of the dissolution medium upon withdrawal. Our previous report with X-ray diffraction analysis confirmed that the PCM may remain in the amorphous state, with 10% *w*/*w* of PCM loading in a similar rice starch film formulation [36]. We speculated that PCM may also present in the amorphous state at a much lower drug loading that allows for better solubilisation when in contact with the dissolution medium. Moreover, this may be due to the inhomogeneous drug distribution, as indicated in the drug loading efficiency study that gave rise to a large variability in the correction made for the drug release study.

Sorbitol was previously reported to reduce the solubility of PCM in an aqueous solution, and the effect is more significant with an increased sorbitol content [48]. Even though the superfluous amounts of sorbitol may seem to reduce the drug solubility, such a reduction is insignificant given a large volume of dissolution medium. The differences in the WAC of rice starch films do not influence the drug release.

## 4. Discussion

The significance of a plasticiser is well recognised in affecting mechanical and physical properties (namely water sorption) of thin film technology, especially in packaging studies [20,55,56,57,58]. Apart from the typical influence of sorbitol on these properties, we observed a remarkable impact of drug content on the plasticising role of sorbitol in the rice starch films, and the effects changed at different sorbitol levels, especially film swelling and flexibility. The EP is not given focus in the discussion due to a strong influence of EB on this parameter. 

**Figure 5:**
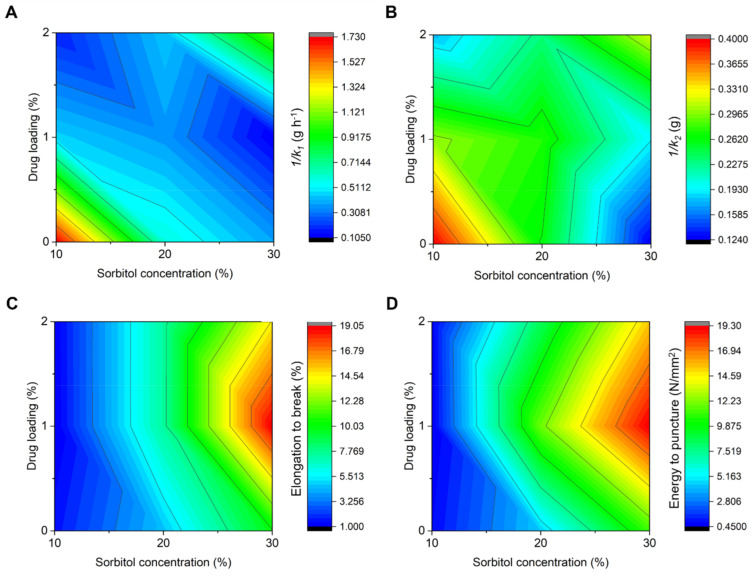
Comparisons of (**A**,**B**) inversed Peleg parameters (1/*k*_1_ and 1/*k*_2_), (**C**) elongation to break and (**D**) energy to puncture of rice starch films.

In order to elucidate the complicated combined effects of sorbitol and drug in the water absorption behaviours (swelling) and mechanical properties, the inversed Peleg parameters and EB are mapped as shown in the colour plots of Figure 5. The Peleg parameters were expressed in the inversed form (1/*k*_1_ and 1/*k*_2_) to allow an easy interpretation of the film swelling and absorption capacity. 

Based on the colour plots of Figure 5A,B, sorbitol and PCM share a similar role in reducing the swelling rate and capacity. PCM and sorbitol have a more intense effect on the swelling rate and WAC, respectively. The slow swelling behaviour was previously mentioned to be related to the strong sorbitol–starch interaction, which limits the water interaction with starch molecules. PCM may react in a similar manner to that of sorbitol to cause a reduction in both swelling-related parameters. In addition, the excellent aqueous solubility of PCM, as shown in the rapid drug release profile, reflects a stronger competition of PCM with starch polymers for water molecules. A lower swelling rate and capacity are thus expected. However, at the highest sorbitol level, the presence of a higher drug content causes both the drug and plasticiser to lose their initial functions in reducing the swelling rate and capacity. The presence of drugs at this loading demonstrated an important interaction with the glucose ring of rice starch, as shown in the FTIR spectrum of the S30P2 film (Figure 3C). Such an interaction may allow for the attraction of water molecules to starch molecules easier. The combination of drugs and plasticisers at the stated concentrations has a unique function that positively affects the swelling behaviour. 

**Figure 6:**
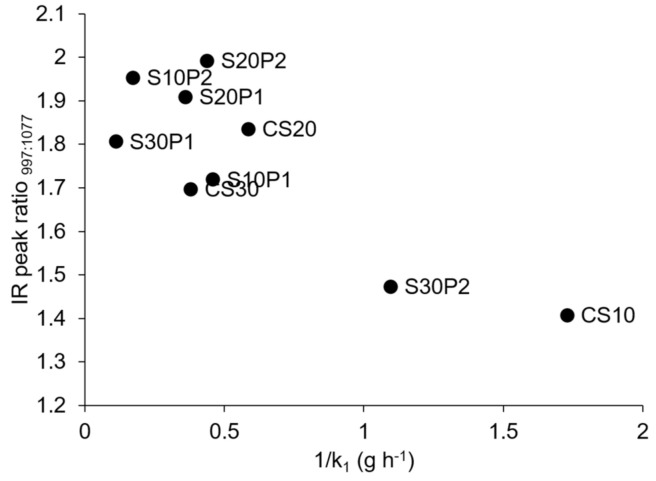
Comparisons of IR peak ratio_997:1077_ and inversed Peleg rate constant.

In addition, we observed a close relationship between the IR peak ratio at 997 cm^−^^1^ (1016 cm^−1^ for S30P2 film) to a constant peak 1077 cm^−^^1^ and the swelling behaviour (1/*k*_1_), as shown in Figure 6. The peak at 997 cm^−^^1^ is sensitive to water content due to the intramolecular hydrogen bonding of the OH groups of the glucose ring [53,59]. A lower peak ratio was observed for films with a higher swelling rate (CS10 and S30P2). This indicates that the presence of PCM in the S30P2 film weakened the intramolecular hydrogen bonding of the glucose ring to allow faster water access to the starch molecules.

For the mechanical aspect, sorbitol was found to have a stronger effect than PCM in increasing EB, as shown in Figure 5C,D. At a low sorbitol level, a reduction in the EB was observed but the effect was subtle. This may be a result of the disturbance of sorbitol-starch interactions by PCM, as discussed previously for swelling behaviour. The establishment of drug–starch interactions constrains the starch polymer chain mobility, and thus, a reduced film flexibility is expected. This can be described as an antiplasticisation effect by PCM. Although the possible interactions between starch and PCM may be established, this is not detected in the FTIR spectra, probably due to a stronger influence from sorbitol–starch interaction. The antiplasticisation effect has been reported in polymeric film systems when the drug or plasticiser is present in a small quantity [33,34,35]. PCM fulfils almost all criteria of antiplasticisers suggested by Jackson Jr. and Caldwell [35] including containing polar atoms such as nitrogen and oxygen and having a glass transition more than −50 °C (glass transition of PCM: 23 °C) [60].

The trend changed at higher sorbitol levels. The excess supply of sorbitol showed a dominant plasticising effect on the film flexibility. This may justify that the additional 10–20% *w*/*w* of sorbitol can overcome the antiplasticising action by a small amount of PCM. The plasticising effect peaked at 20% *w*/*w* of sorbitol and 1% *w*/*w* of PCM. Despite this, a reduced film flexibility was observed with a further increase in both the drug and plasticiser concentrations. In this context, 2% *w*/*w* of PCM is sufficient to counteract the plasticising function from the excess sorbitol content added. This is also indicated in the drug–starch interactions, as evident in the FTIR analysis.

Even though a modification of the sorbitol-plasticising effect on the rice starch films by PCM is found, the differences did not influence the drug release behaviour. This is mainly due to the outstanding aqueous solubility behaviour of PCM, possibly in the amorphous state.

## 5. Conclusions

While sorbitol plasticisation on starch films is well known, this study provides an in-depth understanding of the influence of PCM on modifying the typical plasticising role of sorbitol in rice starch films. Even though the study was carefully designed for the drug crystallinity to be controlled to give room for thorough investigation of sorbitol’s plasticisation behaviour, the influence of the drug, at a low content and most probably in the amorphous state, was still critical in modifying the film’s properties through the antiplasticisation effect. This drug-related antiplasticisation effect is significant at a low sorbitol level, but this effect can be compromised at a higher sorbitol content. Despite this, the antiplasticising state can persist if a higher drug loading is used. These unique changes are well reflected in the film swelling and flexibility behaviours. Noteworthily, such changes in the film swelling and flexibility due to the different combinations of drug and plasticiser contents did not affect the drug release pattern due to the absence of drug crystallinity. Such an interesting finding demonstrated that the interplay between drug and plasticiser contents has a substantial impact on the physicochemical and mechanical properties of the rice starch films. Apart from recognising the crucial role of plasticisers in starch film development, the influence of drugs on the film properties should not be ignored in order to fully comprehend their functions on the buccal film formulations.

## Figures and Tables

**Table 1:** Average thickness (n = 10), water content and drug loading efficiency (n = 3) of all rice starch films (mean ± SD).

Formulation	Film Thickness (mm)	Water Content (%)	Drug Loding Efficiency (%)
**CS10**	0.090 ± 0.014	0.98 ± 0.14	-
**CS20**	0.093 ± 0.019	0.64 ± 0.08	-
**CS30**	0.100 ± 0.014	1.17 ± 0.33	-
**S10P1**	0.080 ± 0.014	0.51 ± 0.36	81.9 ± 5.0
**S20P1**	0.086 ± 0.030	0.89 ± 0.43	93.2 ± 10.6
**S30P1**	0.089 ± 0.017	0.42 ± 0.08	93.2 ± 15.8
**S10P2**	0.077 ± 0.019	0.77 ± 0.15	88.4 ± 29.4
**S20P2**	0.088 ± 0.029	0.74 ± 0.02	71.7 ± 15.1
**S30P2**	0.087 ± 0.020	0.68 ± 0.49	67.2 ± 21.1

**Table 2:** Peleg rate constant (*k*_1_) and capacity constant (*k*_2_) for water absorption kinetic.

Formulation	*k*_1_ (h g^−1^)	*k*_2_ (g^−1^)	R^2^
**CS10**	0.5788	2.5004	0.9972
**CS20**	1.7085	3.8319	0.9700
**CS30**	2.6387	8.0610	0.9134
**S10P1**	2.1822	3.3204	0.9267
**S20P1**	2.7793	3.6524	0.9721
**S30P1**	9.1021	5.1583	0.9673
**S10P2**	5.8184	5.6222	0.9923
**S20P2**	2.2861	4.2975	0.9789
**S30P2**	0.9130	3.1703	0.9475

**Table 3:** Puncture strength, elongation to break and energy to puncture of all rice starch films (n = 3, mean ± SD).

Formulation	Puncture Strength, PS (N/mm^2^)	Elongation to Break, EB (%)	Energy to Puncture, EP (N/mm^2^)
**CS10**	0.43 ± 0.10	1.55 ± 0.54	0.84 ± 0.26
**CS20**	1.04 ± 0.40	4.62 ± 1.13	4.43 ± 2.65
**CS30**	1.62 ± 0.40	9.93 ± 1.14	11.14 ± 3.05
**S10P1**	0.24 ± 0.10	1.05 ± 0.14	0.49 ± 0.24
**S20P1**	1.01 ± 0.51	7.46 ± 1.28	11.88 ± 3.98
**S30P1**	1.41 ± 0.04	19.04 ± 3.10	19.27 ± 2.25
**S10P2**	0.28 ± 0.06	1.04 ± 0.22	0.61 ± 0.18
**S20P2**	1.40 ± 0.39	7.44 ± 0.46	8.48 ± 2.67
**S30P2**	1.60 ± 0.67	14.63 ± 5.06	15.40 ± 3.99
